# Chemoprevention for Breast Cancer

**DOI:** 10.1245/s10434-015-4715-9

**Published:** 2015-07-23

**Authors:** Sandhya Pruthi, Ruth E. Heisey, Therese B. Bevers

**Affiliations:** Division of General Internal Medicine, Department of Internal Medicine, Mayo Clinic, Rochester, MN USA; Department of Family and Community Medicine, University of Toronto, Women’s College Hospital / Princess Margaret Hospital, Toronto, ON Canada; Department of Clinical Cancer Prevention, The University of Texas MD Anderson Cancer Center, Houston, TX USA

## Abstract

**Background:**

Many women at increased risk for breast cancer could benefit from preventive therapy. Preventive therapy options for breast cancer risk reduction have expanded in the last few years to include both selective receptor modulators (tamoxifen and raloxifene) and aromatase inhibitors (anastrozole and exemestane).

**Methods:**

Risk factors that place women at high risk for breast cancer, as well as risk calculation models appropriate for the selection of candidates for preventive therapy, are presented, followed by a review of current guidelines for chemoprevention and results of chemoprevention trials.

**Results:**

The modified Gail model or Breast Cancer Risk Assessment Tool is the most widely utilized risk assessment calculator to determine eligibility for chemoprevention. Women most likely to benefit from preventive therapy include those at high risk under the age of 50 years and those with atypical hyperplasia. Physician and patient barriers limit widespread acceptance and adherence to preventive therapy.

**Conclusions:**

Published guidelines on chemoprevention for breast cancer have been updated to increase awareness and encourage discussion between patients and their physicians regarding evidence-based studies evaluating the benefits of preventive options for women at increased risk for breast cancer. However, even with increasing awareness and established benefits of preventive therapy, the uptake of chemoprevention has been low, with both physician and patient barriers identified. It is prudent that these barriers be overcome to enable high-risk women with a favorable risk-to-benefit ratio to be offered chemoprevention to reduce their likelihood of developing hormone receptor-positive breast cancer.

## Defining Breast Cancer Risk

Defining breast cancer risk incorporates knowledge of individual risk factors known to be associated with increased risk. These risk factors are included in various available risk-calculation models to provide a numeric risk that can be used to help quantify the level of individual risk.^1^

Breast cancer risk factors have historically been described as modifiable versus nonmodifiable factors. Modifiable risk factors in general are associated with lifestyle behaviors and exogenous hormone exposure. These include physical inactivity, increased alcohol consumption, obesity, and use of estrogen and progestin therapies, all of which are associated with increasing breast cancer risk.^2^–^5^ Physicians have an important role in counseling women on the effectiveness of lifestyle modification and avoidance of long-term postmenopausal hormone therapy in the primary prevention of breast cancer. Nonmodifiable risk factors include increasing age, family history, precancerous breast lesions, and reproductive factors (early menarche, late-onset menopause, first live birth after age 30 years, or nulliparity). These risk factors are independently associated with a higher risk of developing breast cancer but it is not known if they are additive for an individual when estimating breast cancer risk.

Breast cancer risk can be categorized as average, high, and very high risk.^6^ In general, a woman having no family history of breast cancer or prior history of a precancerous breast biopsy would be considered at average risk. The lifetime risk for developing breast cancer for an average-risk woman is 12 %. The following criteria are most often used to identify women at high risk: (i) first-degree relative with a breast cancer diagnosis before age 50 years; (ii) history of atypical hyperplasia (AH); (iii) 5-year Gail model risk of ≥1.7 %; (iv) history of lobular carcinoma in situ (LCIS); (v) having received chest radiation between the ages of 10 and 30 years; (vi) increased mammographic breast density; and (vii) International Breast Cancer Intervention Study (IBIS) model (Tyrer–Cuzick) lifetime risk of ≥20 %.^7^–^12^ Breast cancer risk factors and the respective absolute or attributable risk of developing breast cancer are described in Table 1.

**Table 1:** Definition of high risk

Risk factor	Defining high risk
First-degree family member diagnosed at <50 years of age	Twofold risk
Atypical hyperplasia	Cumulative absolute risk is 30 % at 25-year follow-up
Chest radiation between 10 and 30 years of age	40 % lifetime risk
Gail model 5-year risk	Five-year risk ≥1.7 %
Breast density (BI-RADS, D3 or D4)	Women with extremely dense breasts have a twofold increased risk compared with average women
Lobular carcinoma in situ	25 % lifetime risk
International Breast Cancer Intervention Study model (Tyrer–Cuzick model) life-time risk	≥20 % lifetime risk

*BI*-*RADS* Breast Imaging Reporting and Data System, *D3* the breast tissue is heterogeneously dense, *D4* the breast tissue is extremely dense

Women presenting with a strong hereditary predisposition, or known BRCA1 or 2 mutation carriers, are, by definition, considered at very high risk for developing breast cancer. A family history that entails multiple affected relatives with early-onset breast or ovarian cancer over several generations would be an indication to refer to a genetic counselor to discuss the options of genetic testing. The lifetime risk of developing invasive breast cancer for a BRCA mutation carrier is estimated at 40–85 %.^13^ Women with a BRCA mutation should be offered bilateral prophylactic mastectomy (BPM) and risk-reducing salpingo-oophorectomy as these are the only risk-reducing strategies shown to be effective in this population. Those not interested in BPM should have enhanced surveillance with annual mammogram and magnetic resonance imaging, and be offered preventive therapy. The evidence of efficacy of preventive therapy in this population is less compelling.^14^,^15^ Although there is no evidence to support BPM in women who have had thoracic radiation, there is preclinical evidence that tamoxifen decreases the incidence of radiation-induced breast cancer.^16^,^17^

Several complementary risk assessment and calculation tools are available to assist physicians with making decisions regarding preventive therapy, and individualizing risks. These tools incorporate most of the breast cancer risk factors described above and are easily available to the physician at the point of care. When counseling women about preventive therapy, it is recommended that physicians use a shared decision-making approach with women at high or very high risk as they are most likely to benefit from risk-reduction options.^18^,^19^ Women with a history of prior chest-wall radiation age <30 years, or women with a history of LCIS, are considered to be high enough risk to be considered for preventive therapy [National Comprehensive Cancer Network (NCCN) guidelines version 1.2014 Breast Cancer Risk Reduction]. Other women can be assessed for suitability by using a risk assessment tool.

## Determining Eligibility for Preventive Therapy/Risk Assessment Tools

The American Society of Clinical Oncology, the NCCN, the Canadian Task Force on Preventive Health Care, and the US Preventive Services Task Force (USPSTF) advise counseling women ≥35 years of age who are at increased risk for breast cancer regarding available medications to reduce their risk and to offer medication to women at low risk of medication-related side effects (USPSTF B recommendation).^20^–^23^ The Gail model risk calculator is the most widely utilized tool to identify candidates suitable for chemoprevention.^9^,^24^–^26^ The original validated Gail model was updated and modified to become the Breast Cancer Risk Assessment Tool (BCRAT) by the National Cancer Institute and the National Surgical Adjuvant Breast and Bowel Project (NSABP) Biostatistics Center.^27^ The BCRAT includes the following breast cancer risk factors: current age, reproductive history (age at menarche, age at first live birth), history of prior breast disease (number of previous breast biopsies and history of AH), and family history (number of first-degree relatives with breast cancer), with age being the most heavily weighted risk factor.^27^

This model does not include the age of onset of breast cancer in family members, paternal family history, or any family history of ovarian cancer. It is suitable for women ≥35 years of age with no history of ductal carcinoma in situ (DCIS) or LCIS, no prior history of thoracic radiation, and without a strong family history of breast cancer or ovarian cancer suggestive of a genetic predisposition. The model was updated in 2008 to provide adjusted estimates for African American women derived from the Women’s Contraceptive and Reproductive Experiences (CARE) Study and from Surveillance, Epidemiology and End Results (SEER) data, and in 2011 to include Asian and Pacific Islander women using data from the Asian American Breast Cancer Study (AABCS) combined with the SEER database.^25^,^28^

Any woman with a 5-year risk of ≥1.7 % determined by using the Gail model can be considered for preventive therapy. This is the risk estimate utilized for the major breast cancer prevention trials and supported by NCCN guidelines.^21^ Based on risk–benefit tables developed by Freedman et al.^29^ the USPSTF concludes that, in general, women with an estimated 5-year breast cancer risk of ≥3 % are likely to have more benefit than harm from using a selective estrogen receptor modulator (SERM) as chemoprevention, although the balance depends on age, ethnicity, the medication used, and whether or not the patient has a uterus.^23^

In general, women with a history of AH, or women under the age of 50 years, are more likely to benefit from preventive therapy. This is based on the Breast Cancer Prevention Trial (BCPT) data subgroup analysis that demonstrated a significant 86 % risk reduction for women with AH. Furthermore, the evidence supports that women under the age of 50 years are far less likely to incur the harms of therapy seen in women 50 years of age or older.^30^,^31^ Conversely, in many older women the harms of preventive therapy far outweigh the benefits as their risk of adverse effects is greater.

The NCCN Breast Cancer Risk Reduction Panel has adopted the 1.7 % or greater 5-year actuarial breast cancer risk defined by the modified Gail model as the risk threshold for discussion of chemoprevention. This is consistent with eligibility criteria utilized in the NSABP BCPT and the Study of Tamoxifen and Raloxifene (STAR).^30^–^33^Another risk calculation model commonly used is the IBIS or Tyrer–Cuzick model.^11^ It includes BRCA status, height, weight, hormone replacement therapy (HRT) use, age at first live birth, age of onset of cancers in relatives, the presence of ovarian cancer, and second- and third-generation family history on the maternal and paternal side. It is more complex, less accessible to primary care providers, and currently utilized mainly to determine eligibility for enhanced screening with MRI, in addition to mammography, in women with a lifetime risk of breast cancer ≥20 %.

The recently updated American Society of Clinical Oncology guideline on the use of pharmacological interventions for breast cancer risk reduction states that the risk for breast cancer may be determined by the aforementioned BCRAT tool “or other validated models including Tyrer–Cuzick”.^20^

In a head-to-head comparison of the BCRAT and the IBIS model looking at the absolute 10-year risk of breast cancer, the IBIS model showed better discrimination (area under the curve [AUC] for IBIS 69.5 %, 95 % CI 63.8–75.2 versus AUC for BRCAT 63.2 %, 95 % CI 57.6–68.9).^34^

There is no validated model that accounts for breast density, yet it is hoped that one might be developed in the future that will include breast density and be capable of effectively identifying women suitable for both enhanced screening and chemoprevention.^35^

## Preventive Therapy

Tamoxifen and raloxifene, both SERMs, as well as two aromatase inhibitors (AIs), exemestane and anastrozole, have been shown in randomized controlled trials to significantly reduce breast cancer incidence in women at increased risk of the disease.^30^–^33^,^36^,^37^ The SERMs are US FDA approved for this indication in postmenopausal women, although only tamoxifen has been studied and received an indication for breast cancer risk reduction in premenopausal women. The FDA has not approved either of these two AIs for breast cancer risk reduction, and their use in the US is considered off-label. There are a paucity of data on the effectiveness of preventive therapy in women with a history of chest-wall radiation.^38^

### Tamoxifen and Raloxifene

In the landmark BCPT, tamoxifen reduced the risk of breast cancer in both pre- and postmenopausal women at increased risk of the disease by approximately one-half (relative risk [RR] 0.51; 95 % CI 0.39–0.66). Women with AH had a highly significant 86 % breast cancer risk reduction (RR 0.14; 95 % CI 0.03–0.47), whereas women with LCIS, due to the small sample size, had a nonstatistically significant reduction of 56 % (RR 0.44; 95 % CI 0.16–1.06).^30^ Women under the age of 50 years obtained comparable breast cancer risk reduction to women 50 years of age and older. In the 7-year follow-up analysis, the benefits of tamoxifen were shown to persist in women at increased risk of the disease, even after stopping therapy, with a reduction in breast cancer risk of 43 % (RR 0.57; 95 % CI 0.46–0.70). Risk remained decreased by 75 % (RR 0.25; 95 % CI 0.10–0.52) in women with AH, while women with LCIS continued to have a nonstatistically significant risk reduction, now 46 % (RR 0.54; 95 % CI 0.27–1.02).^31^ An updated analysis of the European IBIS-I trial has demonstrated that tamoxifen continues to reduce breast cancer risk at a median of 16 years of follow-up (HR 0.71; 95 % CI 0.60–0.83). The risk of developing breast cancer was similar between years 0–10 (HR 0.72; 95 % CI 0.59–0.88) and after 10 years (HR 0.69; 95 % CI 0.53–0.91).^14^

Tamoxifen is associated with an increased risk of endometrial cancer (RR 2.53; 95 % CI 1.35–4.97; absolute annual risk per 1000: placebo 0.91 vs. tamoxifen 2.30), venous thromboembolic events, including stroke (RR 1.59; 95 % CI 0.93–2.77; absolute annual risk per 1000: placebo 0.92 vs. tamoxifen 1.45), pulmonary embolus (RR 3.01; 95 % CI 1.15–9.27; absolute annual risk per 1000: placebo 0.23 vs. tamoxifen 0.69), deep vein thrombosis (RR 1.60; 95 % CI 0.91–2.86; absolute annual risk per 1000: placebo 0.84 vs. tamoxifen 1.34), cataract development (RR 1.14; 95 % CI 1.01–1.29; absolute annual risk per 1000: placebo 21.72 vs. tamoxifen 24.82), and the need for cataract surgeries (RR 1.57; 95 % CI 1.16–2.14; absolute annual risk per 1000: placebo 3.00 vs. tamoxifen 4.72).^30^ These risks were not significantly different in the 2010 analysis. The serious risks were not significantly increased in women under the age of 50 years, thus identifying a population of women who obtain significant risk reduction benefits without incurring serious harm. Common side effects reported included bothersome hot flashes and vaginal discharge.

The STAR demonstrated that raloxifene was equivalent to tamoxifen in reducing breast cancer risk for postmenopausal women at increased risk of the disease while on therapy.^32^ In the 2010 updated analysis, with a median follow-up of 81 months, benefits with tamoxifen were greater, while the risks were lower with raloxifene. Raloxifene retained 76 % of the effectiveness of tamoxifen in preventing invasive disease. Raloxifene was not associated with an increased risk of uterine cancer risk and has a slightly lower risk of venous thromboembolic events than tamoxifen.^33^ Raloxifene is associated with hot flashes, night sweats, vaginal dryness, and weight gain.

### Aromatase Inhibitors

In the National Cancer Institute of Canada (NCIC) Mammary Prevention 3 (MAP.3) trial, after 35 months of follow-up, exemestane reduced breast cancer risk by 65 % (hazard ratio [HR] 0.35; 95 % CI 0.18–0.70) in high-risk postmenopausal women.^36^ A 53 % reduction in breast cancer risk was seen with anastrozole in the European IBIS-II trial in women at increased risk of breast cancer (HR 0.47; 95 % CI 0.32–0.68).^37^ Data on AIs in women with AH or LCIS are limited. In this subgroup, anastrozole reduced breast cancer risk by 69 % (HR 0.31; 95 % CI 0.12–0.84),^37^ whereas exemestane produced a nonsignificant reduction in the risk of breast cancer by 64 % (HR 0.36; 95 % CI 0.11–1.12).^36^ It is important to note that these analyses are in a very small number of women, limiting the ability to assess the effectiveness of therapies in women with LCIS or AH.

Neither exemestane nor anastrozole were associated with an increased risk of thromboembolic or cardiovascular events, or other cancers. In the MAP.3 trial, although short-term use of exemestane was shown to worsen age-related bone loss in spite of calcium and vitamin D supplementation, long-term follow-up will be needed to assess the effect on fracture risk in a prevention population.^39^ The side effects of exemestane, including vasomotor, sexual, and musculoskeletal symptoms, had limited impact on quality of life.^40^ In addition to vasomotor symptoms, musculoskeletal events (arthralgias, joint stiffness, carpal tunnel syndrome) were more common in the anastrozole arm.^37^

Although it is important that high-risk women be considered for chemoprevention, several barriers have been identified that impact uptake, compliance, and adherence. These include fear of possible side effects of the anti-estrogen therapies, specifically thromboembolic events and an endometrial cancer risk, which may be perceived as outweighing the potential benefits of the pharmacologic therapy on reducing the incidence of breast cancer.^41^–^44^ Furthermore, it is becoming increasingly evident that physicians are encountering barriers to prescribing pharmacologic therapies, including lack of time to effectively counsel patients about available options, knowledge gaps about the risks and benefits of the medications, and challenges with identifying eligible women with a favorable risk-to-benefit ratio who will benefit from the pharmacologic therapy to reduce breast cancer risk.^45^,^46^

## Conclusions

Physicians are strongly encouraged to assess breast cancer risk and appropriately identify high-risk women with a positive risk–benefit ratio eligible for chemoprevention. Communication of the risks and benefits of SERMs and AIs as preventive therapies and shared decision-making approaches are critical to patient uptake and adherence. More widespread utilization of these agents can reduce the incidence of estrogen receptor (ER)-positive breast cancer but will have no impact on ER-negative breast cancer. Future opportunities for breast cancer risk reduction should target hormone-negative, especially triple-negative, breast cancer.
